# Force-Induced Nitric Oxide Promotes Osteogenic Activity during Orthodontic Tooth Movement in Mice

**DOI:** 10.1155/2022/4775445

**Published:** 2022-09-06

**Authors:** Yuqing Sun, Jingfei Fu, Feiran Lin, Shengnan Li, Juan Du, Yi Liu, Yuxing Bai

**Affiliations:** ^1^Department of Orthodontics, School of Stomatology, Capital Medical University, China; ^2^Laboratory of Tissue Regeneration and Immunology and Department of Periodontics, Beijing Key Laboratory of Tooth Regeneration and Function Reconstruction, School of Stomatology, Capital Medical University, China

## Abstract

**Objectives:**

The aim of this study was to investigate the effect of nitric oxide (NO) on orthodontic tooth movement and the regulatory effect on bone formation.

**Design:**

A mouse orthodontic tooth movement model was established to measure the level of releasing NO. Besides, orthodontic tooth movement distance and the bone formation in the tension side of the orthodontic tooth were also analyzed. In vitro, human periodontal ligament stem cells (hPDLSCs) were cultured under tensile force stimulation. The production of NO and the expression level of nitric oxide synthase (NOS) were detected after mechanical stimulation. Furthermore, the downstream cellular signaling pathway regulated by NO was also explored.

**Results:**

The generation of NO steadily increased throughout the orthodontic tooth movement in mice. Orthodontic tooth movement was decreased in the NOS inhibitor group while it was accelerated in the NO precursor group. Force-induced NO promoted the osteogenic differentiation of human hPDLSCs under tensile force stimulation. And force-induced NO in hPDLSCs regulated the PI3K/Akt/*β*-catenin signal pathway.

**Conclusion:**

NO is involved in the regulation of orthodontic tooth movement and promotes bone formation on the tension side of the orthodontic tooth. The PI3K/Akt/*β*-catenin pathway is one of the downstream cell signal transduction pathways of NO in the orthodontic process.

## 1. Introduction

It is known that orthodontic tooth movement (OTM) is a process of bone modification. The balance of bone distraction on the compression side and bone regeneration on the tension side determines the speed of OTM. Historically, several methods have been tested to shorten the orthodontic treatment time including pharmacological, biological, and mechanical stimulation [1–3]. But finding a safe and easy method of reducing orthodontic treatment time is still a primary goal for orthodontists.

Nitric oxide (NO) has been shown to have an effect on bone metabolism. It is a toxic gas, colorless, odorless, and insoluble in water. As the first gas signaling molecule discovered in mammals, NO is produced from L-arginine through activating nitric oxide synthase (NOS). The NO donors have beneficial effects on the growth of osteoblasts and bone formation. In the meanwhile, they decrease the activity of osteoclastic [4, 5]. At the same time, some researchers pointed out that periodontal tissue produces NO under the action of orthodontic force. The precursor of NO accelerates OTM in rats and increases the number of osteoclasts and Howship's lacuna in the compression area, whereas NOS inhibitors that suppress NO production reduce OTM [6–8]. As a result, NO also has a significant impact on OTM.

There have been found three isoforms of NOS: a neuronal form (nNOS), an endothelial form (eNOS), and an inducible form (iNOS) [9]. It has been demonstrated that eNOS mediates bone formation in the tension area, while bone resorption in the compression area is mediated by iNOS. Both eNOS and iNOS seem to be crucial regulators of bone remodeling during the process of OTM, and they distribute mainly in osteocytes [10]. But another study claimed that all NOS isoforms are involved in OTM. Unlike osteocytes, periodontal ligament cells would rather be mechanosensors in the early OTM stage, relating to NO signaling [11]. Human periodontal ligament stem cells (hPDLSCs) are mesenchymal stem cells (MSCs) that are crucial for maintaining the homeostasis of periodontal tissue [12]. Previous evidence suggested that hPDLSCs participate in the process of OTM. Cyclic tensile force (CTF) promoted the expression of osteogenic genes and proteins in hPDLSCs, such as alkaline phosphatase, runt-related transcription factor 2 (RUNX2), osterix, and osteopontin [13]. Moreover, the mechanical strain could stimulate hPDLSCs to differentiate into osteoblasts [14, 15].

Our group has reported that hPDLSCs can produce NO, which has the ability to improve the osteogenic differentiation of PDLSCs [16]. In this study, we focused on the change of NO production of hPDLSCs when exposed to mechanical tension, which simulates the process of OTM. And we also discovered the changes in bone metabolism and the underlying signaling pathway involved in this process.

## 2. Materials and Methods

### 2.1. Animals and Orthodontic Force Application

As experimental animals, male C57BL/6J mice were utilized and purchased from the SPF Biotechnology Cooperation (Beijing). The animals were housed under standard conditions (50-60% humidity, 22-24°C room temperature, standardized mice maintenance diet, and clean water). All animal experiments were performed in accordance with the protocol approved by the institution for animal research (Capital Medical University #2012-x-53).

After intraperitoneal injection of xylazine (20 mg/kg), 24 animals were randomly divided into four groups: control group without OTM, OTM group, OTM+L-arginine group, and OTM+L-NG-monomethyl arginine (L-NMMA) group.

**Figure 1:**
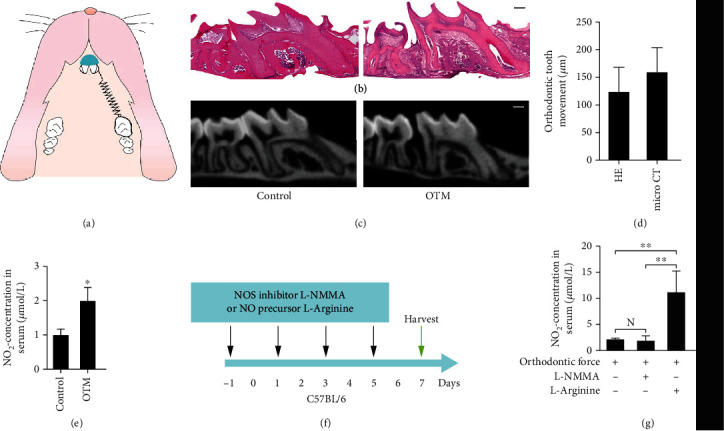
Orthodontic force induces NO production and NOS expression. (a) The orthodontic appliance was fixed between the left maxillary first molar and the incisor. (b) After being applied with orthodontic force, the distal periodontal ligament of the upper first molar widened while the mesial was compressed, scale bar = 200 *μm*. (c) The representative images of micro-CT showed upper first molar moved mesially in the OTM group, scale bar = 200 *μm*. (d) The average distances of OTM were measured in HE and micro-CT images. (e) The concentration of NO_2_^−^ in serum from mice applied with orthodontic force was increased compared with the control group, *n* = 5. (f) Injections of NOS inhibitor and NO precursor were performed every two days starting the day before the application of OTM. (g) The levels of endogenous NO in serum decreased but have no significant difference after interval injections of L-NMMA. The NO levels in serum were significantly upregulated after interval injections of L-arginine, *n* = 5. ^∗^*p* < 0.05 and ^∗∗^*p* < 0.01. Bars and error bars show mean and standard error.

Each mouse with OTM intervention was ligated with a nickel-titanium coil spring (Tomy, Japan), between the maxillary left first molar and both incisors by using a 0.08-inch ligature wire. The spring was elongated for approximately 2 mm to move the maxillary left first molar forward with a stable force of 30 g. Then, the ligature wire was secured on the incisors with primers (Transbond, 3M Unitek, Calif) and Resilience LC Band Cement Syringe (Ortho technology, Florida) (Figure 1(a)). And L-arginine was used as the NO precursor (200 mg/kg, A8094, Sigma-Aldrich), while L-NMMA was used as NO inhibitor (20 mg/kg, M7033, Sigma-Aldrich) to intervene in OTM mice. L-arginine and L-NMMA were injected every two days from the day before the application of OTM (Figure 1(f)). Spring retention was checked daily. The mice were sacrificed 7 days after orthodontic force application. Then, the blood serum and maxillae were collected.

### 2.2. Measurement of OTM Distance

Maxillae were observed, and images were captured under a stereomicroscope. The amount of tooth movement was defined as the shortest distance between the first left maxillary molar's distal-marginal ridge and the second left maxillary molar's mesial-marginal ridge, which was measured by Image-Pro Plus 6.0 (Media Cybernetics). Then, the maxillae were scanned using a micro-CT system (Inveon™, Siemens, Germany) at the resolution of 14.93 *μ*m/pixel. The axial cross-section images were reconstructed from the raw data by Inveon™ Research Workplace software (Siemens). The distance of tooth movement was represented by the distance between the contact points of the first molar and the second molar, repeated by 2 different blinded researchers.

### 2.3. Histological Assessments of Regenerated Periodontal Tissues

Maxillae from the mice were harvested and fixed in 4% paraformaldehyde for 24 h for further experiments. The samples were immersed in a volume of decalcification solution (10% EDTA), and the solution was replaced every 24 hours for 4 weeks. After demineralization, it was dehydrated with ethanol and embedded in paraffin. Semiserial (5 *μ*m) sections were obtained in mesial-distal direction sections of experimental regions and stained with hematoxylin and eosin (HE) for histological analyses.

### 2.4. Immunohistochemical Staining

After the sections were dewaxed by xylene, they were rehydrated with gradient alcohol. Antigen retrieval was carried out using pepsin (Beijing Zsgb-Bio) for 10 minutes at 37°C. Alkaline phosphatase staining was performed with anti-mouse HRP-DAB cells and a tissue staining kit in accordance with the manufacturer's instructions (R&D System, CTS002). Briefly, endogenous peroxidase was consumed with peroxidase blocking reagent for 5 minutes and then incubated for 15 minutes with serum blocking reagents to minimize the nonspecific staining. In addition, the sections were incubated with an avidin blocker for 15 minutes and then incubated for an additional 15 minutes with a biotin blocker. After incubating the sections with the primary antibody against alkaline phosphatase (1 : 300, ab97020, Abcam) at 4°C overnight, the secondary antibodies were later incubated with HRP for an additional 30 minutes. Then, the sections were incubated with HSS-HRP and rinsed several times with PBS. Finally, 3,3′-diaminobenzidine tetrahydrochloride incubated with a chromophore was used for immunohistochemistry, and sections were counterstained with hematoxylin. The image was captured by Aperio ScanScope AT Turbo. Using Image-Pro Plus 6.0, semiquantitative analysis was conducted and the mean optical density (MOD) value was examined.

### 2.5. hPDLSC Isolation and Culture

Every experiment of human stem cells in this study follows the ISSCR “Guidelines for the Conduct of Human Embryonic Stem Cell Research.” The study for human tissue research was conducted according to the protocol which was approved by the Chinese Research Ethical Committee of Capital Medical University (Ethics Committee Agreement, Beijing Stomatological Hospital Ethics Review No. CMUSH-IRB-KJ-PJ-2018-03). Human healthy premolars were acquired with the consent of 5 informed patients (female : male = 2 : 3, aged 12–18 years), and methods for culturing the hPDLSCs have been available previously [17]. Briefly, the periodontal ligaments were scraped gently from the middle one-third of the tooth root, and then, the tissue was digested at 37°C for 1 h using the solution of 3 mg/ml collagenase type I (Worthington Biochemical Corp, Lakewood, NJ, USA) as well as 4 mg/ml dispase (Roche Diagnostics Corp., Indianapolis, IN, USA). Human periodontal ligament cells were cultured in MEM alpha modified Eagle's medium (Invitrogen, Carlsbad, CA, USA), which contained 15% fetal bovine serum (Invitrogen). Meanwhile, it also contained 100 *μ*g/ml streptomycin and 100 *μ*U/ml penicillin as well as 2 mmol/l glutamine (Invitrogen). The culture conditions were 37°C, with CO_2_ concentration of 5% in a humidified incubator, and the culture medium was replaced every three days. The third to the fifth generations of hPDLSCs were used in this study.

### 2.6. Application of Cyclic Tensile Force

Cyclic tensile force was applied to hPDLSCs with Flexcell FX-5000 Strain-Unit (Flexcell Corp., Hillsborough, NC, USA). hPDLSCs (3 × 10^5^/well) were then cultured into six-well and 25 mm flexible-bottomed UniFlex culture plates until 80% confluence. The base of the culture plate forms a collagen-coated silicone membrane. Flexible-bottomed culture plates were attached to a rubber gasket of the Flexcell strain unit (Figure S-e). HPDLSCs were controlled by cyclic tensile force (10% elongation, 0.5 Hz) for 6, 12, and 24 hours. RNA and protein were extracted from hPDLSCs, and the conditioned medium was collected.

### 2.7. Measurement of NO in Blood Serum and Cell Culture Supernatant

In order to measure the levels of NO, cell culture supernatants and mouse serum were collected and carried out the measurement by using a Griess Reagent Kit (Beyotime, China). Absorbance values were then detected at 540 nm with a microplate reader (Bio-Rad Laboratories). The NO_2_^−^ concentration represented the level of NO production and was calculated using a standard curve The NO donors were sodium nitroprusside (SNP; 75 *μ*M, 71778, Sigma-Aldrich), and L-NMMA (1 mM, M7033, Sigma-Aldrich) acted as NOS inhibitors for hPDLSCs. PDLSCs were counted and plated 2 × 10^5^ cells/ml in a six-well plate, and mitomycin C was applied to inhibit the proliferation. After 6 h, 12 h, and 24 h, cell culture supernatants were collected and used for the assay.

### 2.8. Immunofluorescent Staining

For immunofluorescent staining, hPDLSCs were fixed in 4% formaldehyde for 20 min, followed by a blocking buffer (10% normal goat serum) for 30 min. Later, the cells were incubated with primary antibodies CD146 (1 : 100, ab75769, Abcam), CD44 (1 : 100, ab243894, Abcam), and CD45 (1 : 100, ab154885, Abcam) at 4°C overnight and then treated with rhodamine/FITC-conjugated secondary antibodies (1 : 400, A0562, Beyotime). After the final wash, the cells were fixed by a medium containing 4′,6-diamidino-2-phenylindole (P0131, Beyotime). The images were captured by a confocal microscope (AX10, Carl Zeiss, Gottingen, Germany).

### 2.9. Flow Cytometric Analysis

For identification of the MSC phenotype, hPDLSCs were incubated with PBS containing 3% bovine serum albumin with anti-CD44, anti-CD45, and anti-CD146 antibodies (1 : 100, Abcam). After washing with PBS, the cells were incubated with FITC goat anti-rabbit IgG (1 *μ*g/1 × 10^6^ cells, 405404, BioLegend,) in a dark place for 30 min. After another twice washing with PBS, cells were collected followed by fixing with 4% paraformaldehyde for 20 min. And a FACSCalibur flow cytometer was used to analyze the identification of the phenotype of the cell (BD Immunocytometry Systems).

### 2.10. Quantitative Real-Time Polymerase Chain Reaction

Trizol reagent (Invitrogen, USA) was used to extract the total RNA from hPDLSCs. Then, the total DNA was reversely transcribed into cDNA with PrimeScript™ RT Reagent Kit and gDNA Eraser (Takara, Dalian, China). Later, SYBR Premix Ex Taq^TM^ (Takara, Dalian, China) and iCycler iQ Multicolor Real-Time PCR Detection System were used to perform the real-time PCR reactions. The primers synthesized by Sangon Biotech are shown in Table 1.

### 2.11. Western Blotting

Western blotting was carried out in accordance with an existing protocol [16]. Antibodies used include the following: anti-iNOS (1 : 1000, ab178945, Abcam), anti-eNOS (1 : 1000, 32027, CST), anti-RUNX2 (1 : 1000, ab76956, Abcam), anti-PI3K 110*α* (1 : 1000, 4255, CST), anti-PI3K p85*α* (1 : 5000, 13666, CST), anti-Akt (1 : 1000, ab108202 Abcam), anti-p-Akt (1 : 1000, ab384449, Abcam), anti-*β*-catenin (1 : 1000, 8480, CST) antibodies, or anti-active *β*-catenin (1 : 1000, 19807, CST), while GAPDH was detected as a control using anti-GAPDH antibody (1 : 3000, A19056, ABclonal).

### 2.12. Statistical Analysis

The statistical software SPSS 18.0 was used to analyze and process the data. And all the data were examined for normal distribution and homogeneity of variance. Results are expressed as mean ± SEM. For 2-group comparisons, a 2-tailed Student *t*-test was used to test statistical significance; for multiple comparisons, one-way ANOVA with post hoc Bonferroni test was used. For all statistical analyses, *p* < 0.05 was considered statistically significant.

## 3. Results

### 3.1. Orthodontic Force Induces NO Production and NOS Expression In Vivo

To investigate NO production during OTM in vivo, a mouse model with orthodontic force was used to study (Figure 1(a)). After 7 days of force application, HE and micro-CT analysis of OTM showed that the 1^st^ molars were moved mesially. It was observed that the distal periodontal ligament widened while the mesial periodontal ligament was compressed (Figures 1(b)–1(d)). Compared with the control group, the concentration of NO_2_^−^ in the serum of the mice applied with orthodontic force was increased (Figure 1(e)).

### 3.2. Force-Induced NO Regulated the OTM Process and Bone Formation

**Figure 2:**
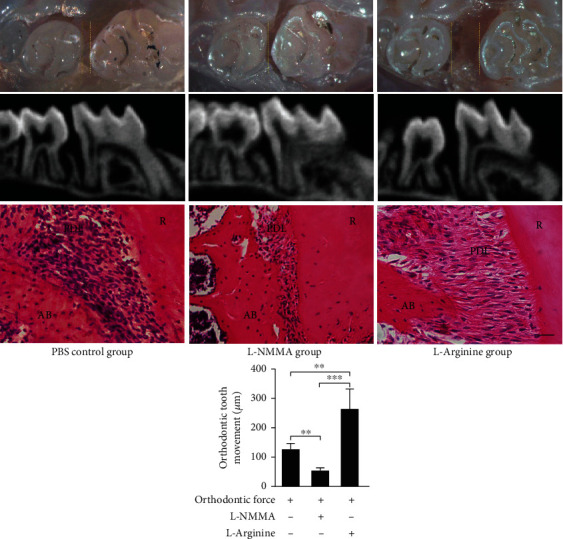
Force-induced NO regulated the OTM process. (a–d) The distance between the upper 1^st^ molar and 2^nd^ molar after 7 d force application was measured by a stereomicroscope (a) and micro-CT (b, d). Inhibition of NOS by L-NMMA injection decreases the subsequent OTM; upregulated NO level by L-arginine injections promotes OTM, (c) HE staining of the periodontal ligament of the distal root of the 1st molar, which is the tension side. L-NMMA injection decreases the subsequent OTM; L-arginine injections promote OTM, scale bar = 50 *μm*. AB: alveolar bone; R: root, *n* > 5. ^∗∗^*p* < 0.01 and ^∗∗∗^*p* < 0.001. Bars and error bars show mean and standard error.

To investigate the effect of force-induced NO production on the OTM process, the NOS inhibitor L-NMMA was injected to suppress it, and the NO precursor L-arginine was injected to increase the endogenous NO production during the OTM process (Figures 1(f) and 1(g)). The distance between the upper 1^st^ molar and 2^nd^ molar was measured by a stereomicroscope (Figure 2(a), Figure S-a) and micro-CT (Figures 2(b) and 2(d)) after 7-day force application. Compared with the control group, the inhibitory effect of L-NMMA on NOS shortened the distance of OTM. In the meanwhile, injection of L-arginine upregulated NO levels and increased the distance of OTM. HE showed the same conclusion (Figure 2(c)).

**Figure 3:**
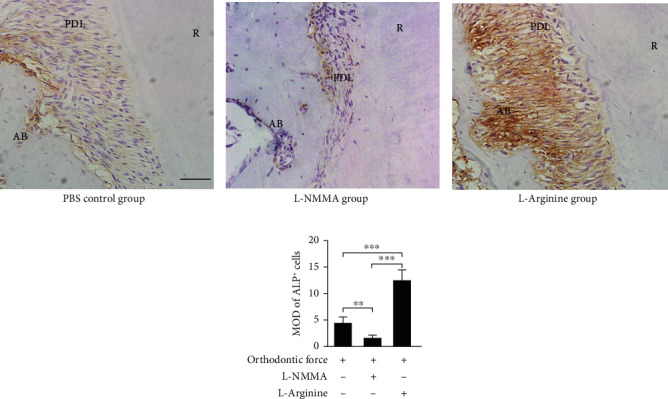
Force-induced NO may regulate bone formation activity on the tension side during OTM. (a–c) Representative immunohistochemical images in the tension side of distal roots. (b, d) L-NMMA injections significantly repressed the expression of alkaline and promoted the expression of alkaline phosphatase in the periodontal ligament. Scale bar = 50 *μm*. ^∗∗^*p* < 0.01 and ^∗∗∗^*p* < 0.001. Bars and error bars show mean and standard error.

Immunohistochemical staining showed that blocking the production of endogenous NO during OTM inhibited the accumulation of alkaline phosphatase-positive hPDLSCs and osteocytes in the periodontal ligament and alveolar bone in the tension area in contrast with the PBS-control group (Figures 3(a), 3(b), and 3(d)). To further confirm the function of NO in OTM, we injected L-arginine to increase the level of NO in mice. The results showed that the accumulation of alkaline phosphatase-positive hPDLSCs and osteocytes in the periodontal ligament and alveolar bone in the tension area was increased compared with the PBS-control group (Figures 3(a), 3(c), and 3(d)).

### 3.3. Cyclic Tensile Force Induces NO Production and NOS Expression In Vitro

**Figure 4:**
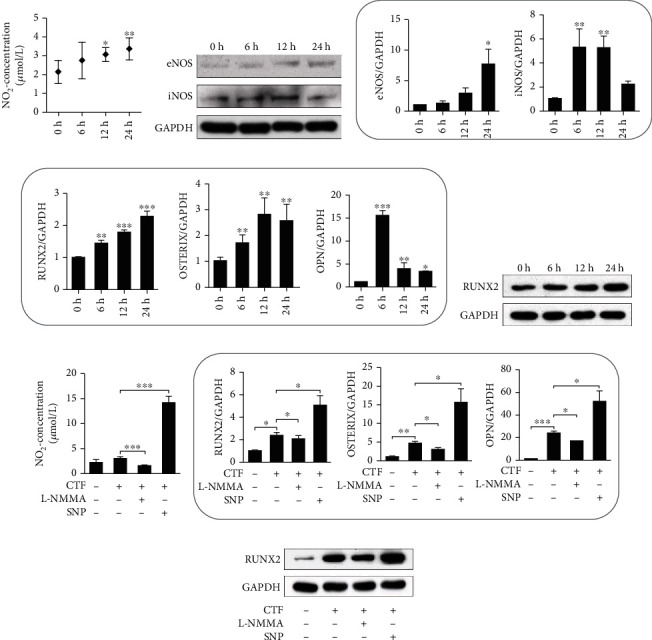
Cyclic tensile force induces NO production and NOS expression to regulate osteogenesis of hPDLSCs. (a) The concentration of NO_2_^−^ secreted by hPDLSCs in the supernatant after CTF application. The concentration of NO_2_^−^ increased significantly after 12 h and 24 h, *n* = 3; (b, c) NOS expression in hPDLSCs was upregulated as assessed by western blotting (b) and RT-PCR. (c) ^∗^*p* < 0.05 and ^∗∗^*p* < 0.01. Bars and error bars show mean and standard error. (d) RUNX2, Osterix, and osteopontin mRNA levels were increased in hPDLSCs after loading, measured by qPCR. (e) The western blot of RUNX2 shows the same tendency. (f) To study the effect of different concentrations of NO, we use NOS inhibitor L-NMMA and NO donor sodium nitroprusside (SNP) during cyclic tensile force application. The concentrations of NO_2_^−^ in cell culture supernatant were examined by the Griess Method. Suppressing NOS by L-NMMA inhibited the increase of NO in human PDLSCs after CTF application. SNP increased NO level in the cell culture supernatant. (g) The results of qPCR showed that RUNX2, Osterix, osteopontin, and mRNA expression was decreased when NO production was suppressed during CTF, and they were increased when NO production was promoted during CTF. (h) The western blot of RUNX2 shows the same tendency. ^∗^*p* < 0.05, ^∗∗^*p* < 0.01, and ^∗∗∗^*p* < 0.001. Bars and error bars show mean and standard error.

Next, we investigated NO production of hPDLSCs which was very essential during bone formation of OTM. Flow cytometry was performed to identify hPDLSCs by expression of CD146, CD44, and CD45 in isolated cells. The percentages of CD146-positive cells and CD44-positive cells were 82.6 and 94.5%, respectively, while the percentage of CD45-positive cells was 0.762%, indicating that the isolated cells were hPDLSCs (Figure S-b, c). Besides, we found that human hPDLSCs expressed eNOS and iNOS assessed by immunostaining (Figure S-d). To investigate the mechanism of NO production, we applied cyclic tension to hPDLSCs to explore the effect of force on NO production in vitro (Figure S-e). It has been proved that hPDLSCs produce NO without force stimulation. The concentration of NO_2_^−^ in culture supernatant of hPDLSCs was examined, and it increased significantly after 12 h and 24 h (Figure 4(a)). Western blot results showed that eNOS and iNOS protein expressions were upregulated, and qPCR results showed the same tendency (Figures 4(b) and 4(c)). These data indicated NO production was promoted in hPDLSCs by CTF, which may have implications for biological function.

### 3.4. Force-Induced NO May Regulate Bone Formation Activity on the Tension Side during OTM

We hypothesized that bone formation on the tension side during OTM might be regulated by force-induced NO via osteogenic differentiation of hPDLSCs. Previous studies have shown that circulation tension promoted osteogenic differentiation of hPDLSCs. We treated hPDLSCs with CTF for 0 h, 6 h, 12 h, and 24 h. RUNX2, Osterix, and osteopontin mRNA levels increased after measurement by qPCR (Figure 4(d)). The western blot results of RUNX2 showed the same trend (Figure 4(e)). To investigate the effect of different concentrations of NO on osteogenesis, hPDLSCs were treated with L-NMMA to reduce NO_2_^−^ concentration by hPDLSCs, and SNP increased NO_2_^−^ concentration in vitro during cyclic tension application (Figure 4(f)). The qPCR results showed that RUNX2, Osterix, and osteopontin mRNA expression was decreased when NO production was suppressed during CTF, and they were increased when NO production was promoted during CTF (Figure 4(g)). Western blot of RUNX2 showed the same tendency (Figure 4(h)).

### 3.5. Force-Induced NO in hPDLSCs May Regulate the PI3K/Akt Signal Pathway

**Figure 5:**
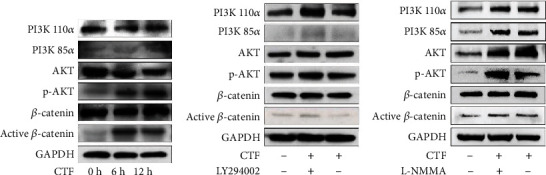
Force-induced NO in PDLSCs may be regulated by iNOS via the PI3K/Akt signal pathway. (a) Western blot indicating that PI3K P85*α*, p-Akt, and active *β*-catenin levels were significantly increased after 12 h CTF. (b) While PI3K inhibitor LY294002 reduced the upregulation of these markers. (c) The NO inhibitor L-NMMA also suppressed the upregulation of the PI3K/Akt pathway treated by CTF.

To investigate how force-induced NO affects the osteogenesis of hPDLSCs, we analyzed the NO downstream pathway PI3K/Akt. The expression levels of p-PI3K, p-Akt, and active *β*-catenin were upregulated after stimulation of hPDLSCs (Figure 5(a)). Then, we treated hPDLSCs with the PI3K inhibitor LY294002. The expression levels of p-PI3K, p-Akt, and active *β*-catenin were downregulated (Figure 5(b)). Besides, we also treated the hPDLSCs with the NO inhibitor L-NMMA and found that L-NMMA suppressed the promotion of the PI3K/Akt pathway in hPDLSCs treated with CTF (Figure 5(c)).

## 4. Discussion

In this study, our experimental results demonstrate that force-induced endogenous NO promotes osteogenic differentiation of hPDLSCs to regulate the OTM process. First of all, we found that the concentration of NO_2_^−^ in mouse serum increased during orthodontic force application. And blocking endogenous NO or increasing NO levels can inhibit or promote OTM and affect osteogenic activity. In addition, the expression of alkaline phosphatase in the periodontal ligament induced by orthodontic force was increased, which promoted the differentiation of osteoblasts in the alveolar bone. Therefore, we speculated that NO induced by hPDLSCs promotes osteogenesis. To test this hypothesis, we confirmed in vitro that mechanical force indeed promotes the expression of NOS in hPDLSCs, and the levels of NO production are also increased. Meanwhile, force-induced endogenous NO in hPDLSCs promoted RUNX2 expression and osteogenic differentiation of hPDLSCs under mechanical stimulation. These results indicated that force-induced endogenous NO promotes the osteogenic activity of PDLSCs, which contributes to the maintenance of the homeostasis of the alveolar bone during OTM.

NO was found to be an essential gas signaling molecule in mammalian cells [18, 19]. It has a variety of effects on bone, including bone resorption, bone healing, and bone formation. Lack of eNOS in mice inhibits osteoblast differentiation, which leads to impaired bone formation [20]. At present, the role of NO in OTM has been reported. Using the intervention of L-arginine (NO precursor) and L-NMMA (nitric oxide synthase inhibitor), they demonstrated that NO can promote OTM, but the specific mechanism has not been elucidated [21]. The present study confirmed this conclusion, finding that force-induced endogenous NO can regulate the process of OTM, and then, we explored the role of NO in it.

Our previous study found that hPDLSCs expressed iNOS and eNOS and produced NO, while L-NMMA attenuated their osteogenic differentiation capacity [16]. And other studies also proved that NO was readily detected in the medium in the presence of MSCs [22]. Besides, the expression of iNOS was suggested to relate to the immunosuppressive capacity of MSCs [23]. It has been also reported that NO production is increased in hPDLSCs through the process of osteogenic differentiation [16]. Therefore, we wondered whether NO production induced by CTF could regulate osteogenic differentiation in hPDLSCs. The results of this study demonstrate that endogenous NO production of hPDLSCs is increased by mechanical stimulation. NO promotes alkaline phosphatase production, induces bone formation, and regulates orthodontic bone remodeling. In addition, when NO synthase inhibitor L-NMMA was used to reduce NO levels, the expression levels of RUNX2, Osterix, and osteopontin were downregulated in hPDLSCs under mechanical stimulation. This result is agreed with previous researches that NO regulates differentiation of osteoblast in vitro and induces osteogenesis in vivo [16, 24]. And it is the first to demonstrate that mechanical force-induced NO production regulates the osteogenic differentiation of hPDLSCs in vitro.

The PI3K/AKT pathway, which regulates multiple cellular functions such as cell proliferation, differentiation, and apoptosis, is mutated in human cancers [25, 26]. Recently, the PI3K/Akt signaling pathway was identified to regulate OTM and periodontium remodeling [24]. We confirmed that OTM could activate the PI3K/Akt/*β*-catenin signaling pathway in hPDLSCs. And the results of L-NMME intervention indicated that NO secreted by hPDLSCs could play a role through the PI3K/Akt/*β*-catenin signaling pathway.

Some scholars have reported that NO accelerates OTM by increasing the number of osteoclasts and promoting bone resorption during OTM. However, NO was found to accelerate OTM by increasing the numbers of osteoclasts and promoting bone resorption during OTM. But osteoclastic activity was not investigated in our experiment, which should be examined by tartrate-resistant acid phosphatase staining and immunohistochemical staining in the future. NO has a biphasic regulation to bone remodeling with a dependency on concentrate. Our research and previous reports may indicate that NO precursors promote bone formation and bone resorption during OTM. The difference between NO production and NOS expression between the tension side and the pressure side remained to be clarified, which may cause different biologic activities. Besides, the producers of NO during inflammatory events are macrophages and endothelial cells [27], but we did not discuss the effects of these cells. This study can only prove that the production of NO, which is secreted by different cells, could affect the osteogenic differentiation of hPDLSCs in vivo.

## 5. Conclusions

The functional role of NO in OTM has not been fully elucidated. Our study indicates that NO is required to regulate the OTM process. The reduction of NO levels leads to shortened OTM distances. NO facilitates the osteogenic activity of the tension area by promoting osteogenic differentiation of PDLSCs. It is expected that with the deepening of the understanding of the role of NO in OTM, an accelerated OTM treatment method based on N**O** donors may be brought.

## Figures and Tables

**Table 1:** The primers synthesized by Sangon Biotech in the study.

	Forward	Reverse
GAPDH	5′-AACCTGCCAAATATGATG AC-3′	5′-ATACCAGGAAATGAGCTT GA-3′
RUNX2	5′-TCCACACCATTAGGGACCATC-3′	5′-TGCTAATGCTTCGTGTTTCCA-3′
Osterix	5′-GCCATTCTGGGCTTGGGTA-3′	5′-TGTGGCAGGGCCAGAGTCTA-3′
Osteopontin	5′-CATCACCTGTGCCATACCAG-3′	5′-GCCACAGCATCTGGGTATTT-3′
iNOS	5′-CACCATCCTGGTGGAACTCT-3′	5′-TCCAGGATACCTTGGACCAG-3′
eNOS	5′-GATGCTCCCAACTTGACCAT-3′	5′-TAGGTCTTGGGGTTGTCAGG-3′

## Data Availability

All data analyzed during the current study are included in this published article. Please contact the corresponding author for data requests.

## Supplementary Materials

Supplementary Materials The supplementary file is the supplementary data related to results that might be of interest to readers.
