# Supplementary material for: Force-Induced Nitric Oxide Promotes Osteogenic Activity during Orthodontic Tooth Movement in Mice
Source: Stem Cells Int. 2022 Sep 6;2022:4775445. doi: 10.1155/2022/4775445 (PMC9470363; doi:10.1155/2022/4775445)
Supplement: Supplementary Materials — The supplementary file is the supplementary data related to results that might be of interest to readers. [file 4775445.f1.docx]

### Supplemental figure


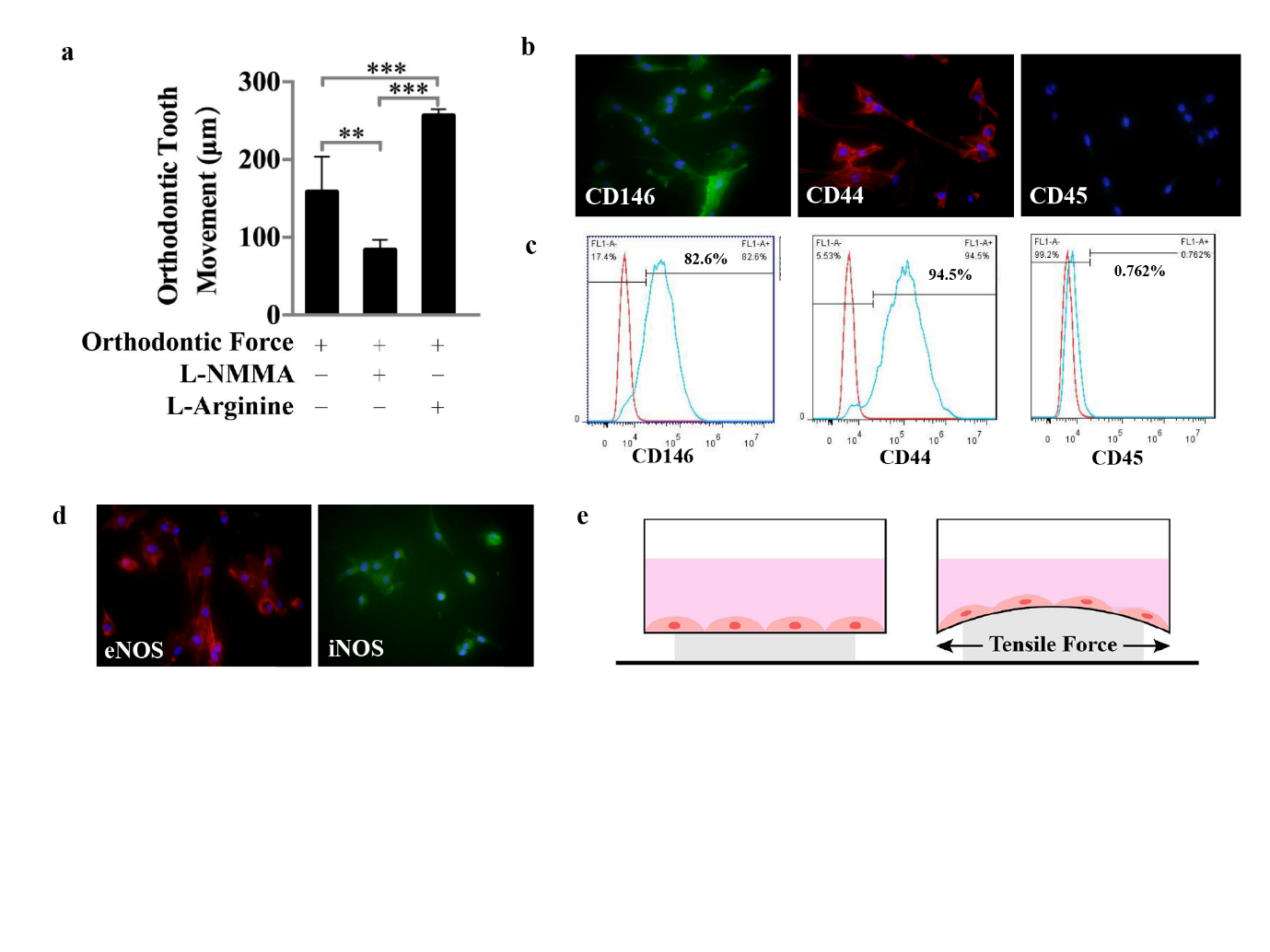


**(a**) Distance statistics of HE staining. L-NMMA injection decreases the subsequent OTM; L-Arginine injections promote OTM. **(b)** As Immunofluorescent staining results showed hPDLSCs expressed CD146 and CD44, but little CD45; scale bar=20μm. **(c)** Flow cytometric analysis demonstrated that hPDLSCs expressed CD146 (82.6%), CD44 (94.5%), but little CD45 (0.762%). **(d)** hPDLSCs expressed eNOS and iNOS, as assessed by immunofluorescent staining. (**e**) The schematic diagram of CTF. The basement membrane of the cell culture plate is deformed and increased in the area, and the cells attached to the membrane are stretched and forced. n>5. ** p<0.01, *** p<0.001, Bars and error bars show mean and standard error.
